# Bazedoxifene enhances the anti-tumor effects of cisplatin and radiation treatment by blocking IL-6 signaling in head and neck cancer

**DOI:** 10.18632/oncotarget.11464

**Published:** 2016-08-22

**Authors:** Arti Yadav, Bhavna Kumar, Theodoros N. Teknos, Pawan Kumar

**Affiliations:** ^1^ The Ohio State University Comprehensive Cancer Center, Columbus, OH 43210, USA; ^2^ Department of Otolaryngology-Head and Neck Surgery, The Ohio State University, Columbus, OH 43210, USA

**Keywords:** bazedoxifene, IL-6, HNSCC, chemoresistance, cancer stem cell

## Abstract

Recent studies have shown that IL-6 signaling plays an important role in the aggressive and metastatic phenotype of head and neck squamous cell carcinoma (HNSCC). Therefore, we hypothesized that targeting of IL-6 signaling in HNSCC could enhance the therapeutic efficacy of standard chemoradiation treatment. We used both *in vitro* and *in vivo* models to test the efficacy of Bazedoxifene (BZA), a drug that was originally developed as a newer-generation selective estrogen receptor modulator (SERM) for the treatment of postmenopausal osteoporosis. Recently, BZA was also shown to exhibit potent anti-cancer effects that were both estrogen receptor (ER)-dependent and ER-independent. Our results suggest that BZA inhibits IL-6 signaling by disrupting IL-6R/gp130 protein-protein interactions. BZA treatment of CAL27-IL-6 (IL-6 overexpressing cells) or UM-SCC-74A (naturally expressing high levels of IL-6) significantly inhibited cell proliferation, migration and colony formation ability in a dose-dependent manner. In addition, BZA significantly decreased IL-6-mediated tumorsphere formation by markedly reducing nanog expression. BZA treatment also markedly reduced chemo and radioresistance in head and neck cancer cells by downregulating ERCC-1, XRCC-1 and survivin expression. In a SCID mouse xenograft model, BZA significantly enhanced the anti-tumor effects of cisplatin and radiation treatment with no added systemic toxicity. Furthermore, combination treatments significantly decreased tumor metastasis, pSTAT3 expression and nanog expression, *in vivo*. Taken together, our results suggest that targeting IL-6 signaling with bazedoxifene could be an effective treatment strategy for the treatment of HNSCC patients.

## INTRODUCTION

Head and neck squamous cell carcinoma (HNSCC) is the 8^th^ leading cancer worldwide with almost 650,000 new cases diagnosed every year and 350,000 cancer-related deaths annually [1–3]. Although advancements in the anti-cancer treatments including surgery, radiation and chemotherapy have increased the local control of HNSCC, the overall survival rates have not improved significantly over the last three decades [4, 5]. Five year survival rates for patients with early stage localized head and neck cancers are more that 80% but drop to 40% when the disease has spread to the neck nodes, and to below 20% for patients with distant metastatic disease [4]. Therefore, there is a need to develop novel therapeutic strategies that are more effective and have fewer side effects than currently used treatment regimens.

IL-6 is one of the main chemokines present in serum samples of cancer patients and elevated IL-6 levels have been shown to be an independent predictor of tumor recurrence, poor survival and tumor metastasis in a number of malignancies including breast, prostate and head and neck cancers [6–10]. IL-6 was initially identified and cloned as B-cell stimulatory factor-2 [11–13]. At the same time a number of other molecules (IFN-β2, plasmacytoma growth factor and hepatocyte-stimulating factor) were independently cloned and found to be identical to IL-6 [14–17]. IL-6 is produced by a wide variety of cell types including immune cells (macrophages, dendritic cells and B-cells), endothelial cells and tumor cells [18–21]. IL-6 is a pleiotropic cytokine that is involved in a number of cellular functions including, immune response, cell proliferation, cell survival and cell migration [18, 22–24]. IL-6 exerts its biological effects predominantly through binding to IL-6 receptor-α (IL-6Rα) on the cell surface [25]. IL-6 binding to its receptor in turn induces conformational changes leading to the formation of IL-6/IL-6Rα/gp130 hexameric complex (a gp130 homodimer plus IL-6/IL-6Rα hetrodimers) [26–28]. This complex then recruits Janus (JAK) kinases and phosphorylates them [29]. Activated JAKs, phosphorylate cytosolic STAT3 which then translocate to the nucleus to function as a transcription factor [30]. In addition to the activation of JAK/STAT3 pathway, IL-6 also mediates its signaling by activating PI3K/AKT, RAS/MAPK and Wnt signaling pathways [18, 31, 32]. IL-6 role in tumor development was initially described in pristane-induced peritoneal plasma cell tumors (PCT), where it was shown that IL-6 is the principal factor promoting the growth of PCT [33, 34]. In head and neck cancers, IL-6 expression and its role in STAT3 activation and tumor growth has been extensively documented [8–10, 35–37]. Recently, we have shown that IL-6 promotes tumor metastasis by inducing epithelial-mesenchymal transition (EMT) [18]. A number of studies have also highlighted the role of IL-6 in the acquisition of chemo and radioresistance and stem cell phenotype in cancer cells [38–41]. Therefore targeting IL-6 signaling is a potential therapeutic strategy for the treatment of HNSCC.

Bazedoxifene acetate (BZA) is a newer generation selective estrogen receptor modulator (SERM) that is currently used in clinics to treat postmenopausal osteoporosis [42–44]. Further studies have shown that BZA acts as an estrogen antagonist in the breast. In MCF-7 cells, a breast cancer cell line, BZA significantly inhibited estradiol-stimulated proliferation [45]. In a separate study, BZA was shown to inhibit the proliferation of both hormone-dependent and hormone independent breast cancer cells by downregulating Cyclin D1 [46]. In addition, BZA also inhibited the growth of tamoxifen-sensitive and tamoxifen-resistant tumors in a mouse xenograft model [47]. Huameng et al recently showed that BZA is able to block IL-6-mediated STAT3 activation [48]. In this study, we used both *in vitro* and *in vivo* models to examine the efficacy of BZA either a single agent or in combination with cisplatin or radiation for the treatment of head and neck cancer. Our results suggest that BZA inhibits head and neck cancer cell proliferation by blocking IL-6 signaling. BZA treatment also significantly reversed chemo and radioresistance in tumor cells by downregulating XRCC-1, ERCC-1 and survivin. In addition, BZA markedly decreased IL-6-mediated tumor cell migration and cancer stem cell phenotype by inhibiting FAK activation and decreasing the expression of nanog. In a SCID mouse xenograft model, BZA significantly decreased tumor growth and tumor metastasis.

## RESULTS

### Bazedoxifene (BZA) significantly inhibited HNSCC tumor cell proliferation and colony formation

**Figure 1:**
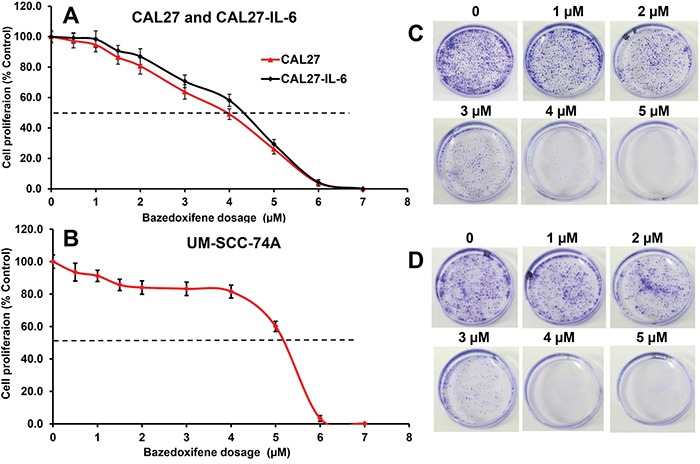
Bazedoxifene inhibits tumor cell proliferation and colony formation in a dose-dependent manner **A-B**. CAL27 (A), CAL27-IL-6 (A) and UM-SCC-74A (B) cells were treated with different concentrations of bazedoxifene and cell proliferation assessed by MTT assay. The percentage cell proliferation for each treatment group was calculated by adjusting the untreated group to 100%. **C-D**. CAL27-IL-6 (C) or UM-SCC-74A (D) cells were treated with different concentrations of bazedoxifene and colony formation was examined by culturing tumor cells in 3.5 cm petri-dishes. After 10 days, colonies were fixed with ice-cold methanol and stained with 0.5% crystal violet solution.

We selected CAL27, CAL27-IL-6 and UM-SCC-74A HNSCC cell lines to examine the effect of BZA on tumor cell proliferation and colony formation. We have recently shown that overexpression of IL-6 in CAL27 cells (CAL27-IL-6) significantly enhances tumor growth and tumor metastasis [18]. In addition to CAL27-IL-6 cells, we selected UM-SCC-74A (a naturally high IL-6 producing cell line) that is highly metastatic and chemo & radioresistant [49, 50]. BZA treatment of CAL27-IL-6 cells resulted in 2%, 9%, 29%, 42%, 71% and 96% growth inhibition at 1, 2, 3, 4, 5 and 6 μM doses, respectively with IC_50_ of 4.5 μM (Figure 1A), whereas parental CAL27 cells were slightly more sensitive to BZA treatment with IC_50_ of 4 μM (Figure 1A). BZA treatment of UM-SCC-74A cells resulted in 9%, 14%, 17%, 19%, 40% and 97% growth inhibition at 1, 2, 3, 4, 5 and 6 μM doses, respectively with IC_50_ of 5.2 μM (Figure 1B). In addition, BZA was very potent in inhibiting tumor cell colony formation in both CAL27-IL-6 and UM-SCC-74A cells (Figure 1C-1D).

### BZA mediates its anti-tumor effects by blocking IL-6 signaling

**Figure 2:**
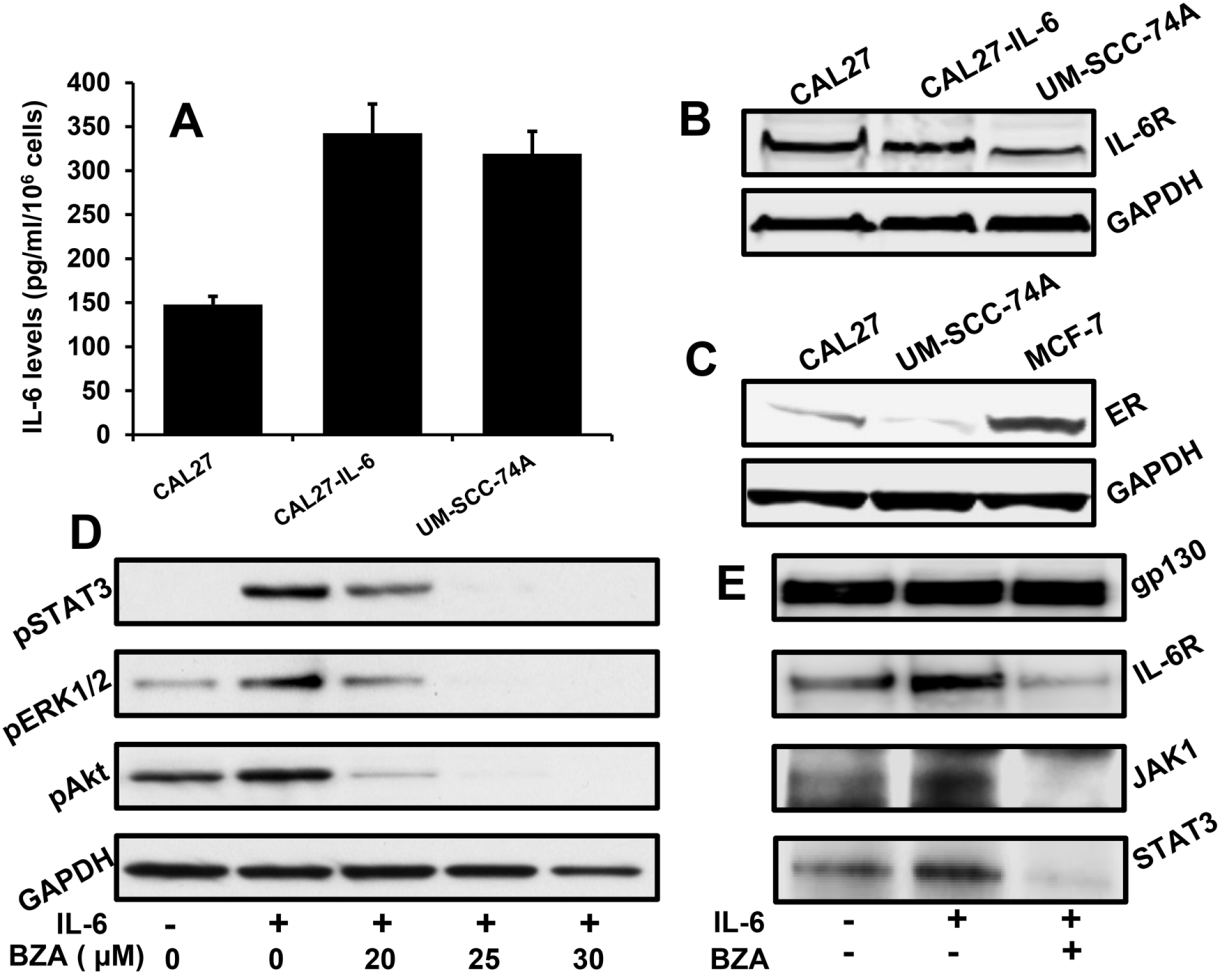
Bazedoxifene inhibits IL-6 signaling by blocking IL-6R and gp130 interactions **A**. CAL27, CAL27-IL-6 and UM-SCC-74A cells were cultured in 6 cm dishes. After 72 hours, culture supernatants were collected and IL-6 levels assayed by ELISA (R&D Systems). **B**. Whole cell lysates for CAL27, CAL27-IL-6 and UM-SCC-74A cells were Western blotted for IL-6R expression. Equal protein loading was verified by stripping the blot and reprobing with GAPDH. **C**. Whole cell lysates for CAL27 and UM-SCC-74A cells were Western blotted for ER expression. Equal protein loading was verified by stripping the blot and reprobing with GAPDH. **D**. CAL27 cells were treated with different concentrations of bazedoxifene (BZA) in serum free medium for 2 hours and then treated with IL-6 (50 ng/ml). After 30 minutes, whole cell lysates were prepared and Western blotted for pSTAT3, pERK1/2 and pAkt. Equal protein loading was verified by stripping the blot and reprobing with GAPDH. **E**. CAL27 cells were treated with IL-6 in the presence or absence of bazedoxifene (25 μM). After 30 minutes, whole cell lysates were prepared and gp130 immunoprecipitated. Proteins bound (IL-6R, JAK1 and STAT3) to gp130 were analyzed by Western blotting.

BZA has been extensively studied as selective estrogen receptor modulator and recently it has also been shown to mediate anti-tumor effects [43, 45, 47]. In this study, we examined if BZA could mediate its anti-tumor effects by blocking IL-6 signaling. We first characterized CAL27, CAL27-IL-6 and UM-SCC-74A cell lines for IL-6 and IL-6R expression. CAL27, CAL27-IL-6 and UM-SCC-74A showed 146 pg/ml, 345 pg/ml and 319 pg/ml of IL-6 levels, respectively (Figure 2A). In addition, all of these cells lines expressed moderate to high levels of IL-6R (Figure 2B). We next examined ER expression in CAL27 and UM-SCC-74A cells. MCF-7 (breast cancer line) was used as a positive control for ER expression. As compared to MCF-7 cells, CAL27 cells expressed low to moderate ER levels, whereas UM-SCC-74A cells had very low ER expression (Figure 2C). However, both of the HNSCC cell lines are not dependent on ER signaling for survival or growth. BZA treatment markedly inhibited IL-6-mediated STAT3, Akt and ERK1/2 phosphorylation in a dose-dependent manner in CAL27 cells (Figure 2D). We next examined if BZA inhibited IL-6 signaling by blocking IL-6R/gp130 complex formation. In our immunoprecipitation experiments, BZA treatment markedly reduced IL-6R binding to gp130 (Figure 2E). In addition, BZA treatment also blocked JAK1 and STAT3 binding to gp130 (Figure 2E).

### BZA significantly reversed cisplatin and radiation resistance in HNSCC cells

**Figure 3:**
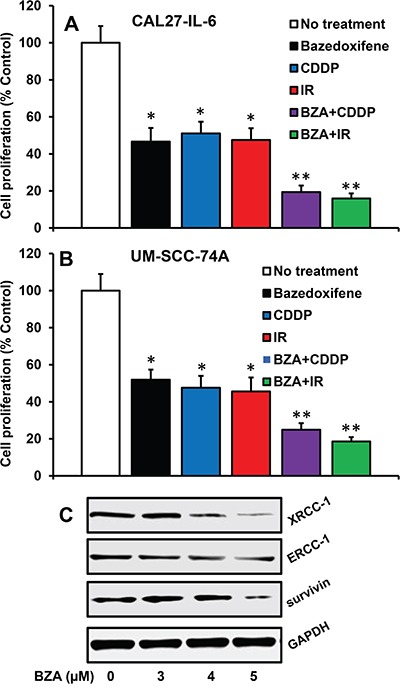
Bazedoxifene reverses cisplatin and radiation-resistance by downregulating XRCC-1, ERCC-1 and survivin **A-B**. CAL27-IL-6 (A) cells orUM-SCC-74A cells (B) were treated with the respective IC_50_ dose of bazedoxifene (BZA), cisplatin (CDDP) or radiation (IR) either alone or in combination and cell proliferation assessed by MTT assay. The percentage cell proliferation for each treatment group was calculated by adjusting the untreated group to 100%. **C**. CAL27 cells were treated with IL-6 in the presence or absence of bazedoxifene (BZA). After 24 hours, whole cell lysates were prepared and Western blotted for ERCC-1, XRCC-1 and survivin. Equal protein loading was verified by stripping the blot and reprobing with GAPDH.

We next examined if blockade of IL-6 signaling by BZA could reverse chemo and radioresistance in head and neck cancer cells. Indeed, BZA treatment significantly reversed chemo and radioresistance in both CAL27-IL-6 and UM-SCC-74A cells (Figure 3A-3B). We have recently shown that ERCC-1, XRCC-1 and survivin are key molecules that regulate chemo and radioresistance in HNSCC [49, 51]. Our results from this study show that BZA treatment is highly effective in reducing XRCC-1, ERCC-1 and survivin expression in tumor cells (Figure 3C).

### BZA treatment significantly decreased tumor cell motility and stemness phenotype in HNSCC cells

**Figure 4:**
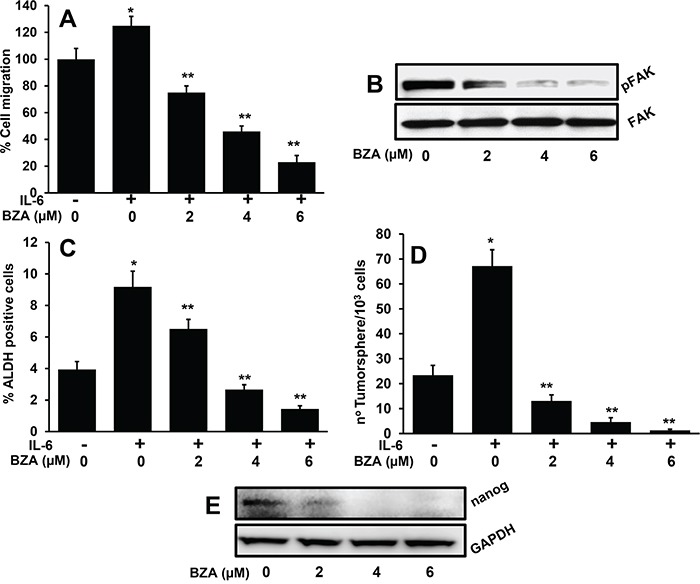
Bazedoxifene significantly decreases IL-6-mediated cell migration, ALDH expression and tumorsphere formation **A**. Tumor cell motility was examined by scratch assay. Each assay was photographed and distances between the migrating cell edges were quantified and percentage cell migration was calculated. *, represents a significant difference (p<0.05) as compared to no treatment group and **, represents a significant difference (p<0.05) as compared to IL-6 treatment group. **B**. CAL27 cells were treated with IL-6 in the presence or absence of bazedoxifene (BZA). After 24 hours, whole cell lysates were prepared and Western blotted for pFAK. Equal protein loading was verified by stripping the blot and reprobing with GAPDH. **C**. CAL27 cells were treated with IL-6 and different concentrations of bazedoxifene (BZA). After 24 hours, cells were stained for ALDH and analyzed by flow cytometry. *, represents a significant difference (p<0.05) as compared to no treatment group and **, represents a significant difference (p<0.05) as compared to IL-6 treatment group. **D**. CAL27 cells were cultured in ultra-low binding plates in the presence of IL-6 and different concentrations of bazedoxifene (BZA). After 10 days, tumorsphere (>50 μM) were counted. *, represents a significant difference (p<0.05) as compared to no treatment group and **, represents a significant difference (p<0.05) as compared to IL-6 treatment group. **E**. CAL27 cells were treated with IL-6 in the presence or absence of bazedoxifene (BZA). After 24 hours, whole cell lysates were prepared and Western blotted for nanog. Equal protein loading was verified by stripping the blot and reprobing with GAPDH.

We had recently shown that IL-6 promotes tumor cell migration by regulating the FAK activation [18]. Results from this study show that BZA treatment significantly decreased tumor cell migration in a dose-dependent manner (Figure 4A). In addition, we also observed a dose-dependent inhibition of FAK phosphorylation (Figure 4B). IL-6 signaling plays a critical role in cancer stem cell phenotype [40, 52]. We next examined if BZA treatment could decrease IL-6-mediated stemness phenotype in cancer cells. Indeed, BZA treatment significantly decreased the proportion of ALDH positive cells (Figure 4C) and the number of tumorsphere formed (Figure 4D). Nanog is one of the key stem cell transcription factors that plays an important role in cancer stem cell phenotype [53, 54]. BZA treatment significantly decreased nanog expression in cancer cells as well (Figure 4E).

### BZA inhibits tumor growth in a dose-dependent manner

**Figure 5:**
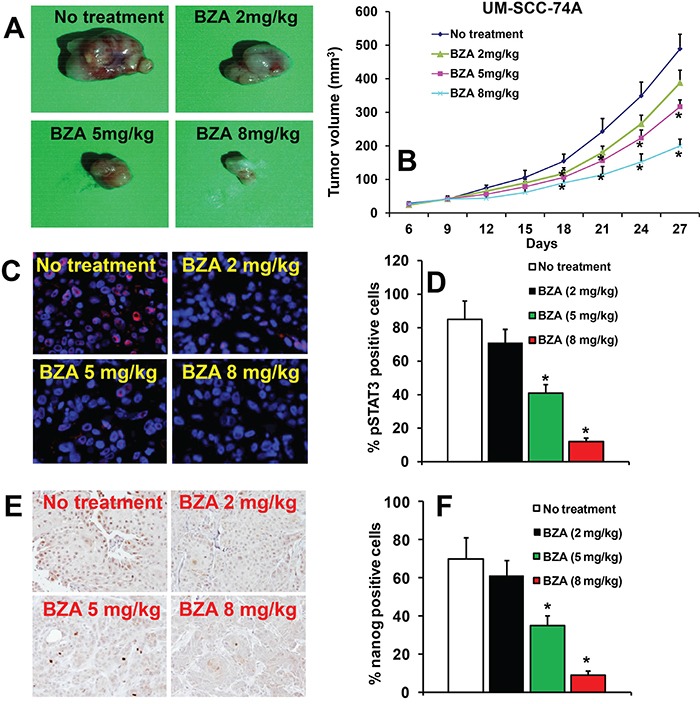
Bazedoxifene inhibits tumor growth in a dose-dependent manner **A-B**. Animals bearing UM-SCC-74A tumors were treated with Bazedoxifene (BZA) at different doses (2 mg/kg, 5 mg/kg or 8 mg/kg) as described in methods. (A) Representative photomicrographs of tumors from untreated, BZA 2 mg/kg, BZA 5 mg/kg and BZA 8 mg/kg groups. (B) Tumor growth curves for UM-SCC-74A tumors treated with different doses of BZA. **C-F**. pSTAT3 (C-D) and nanog levels (E-F) in UM-SCC-74A tumors at the end of the *in vivo* experiments. *, represent a significant difference (p<0.05).

To confirm the anti-tumor effects of BZA, *in vivo*, we used a SCID mouse xenograft model. Animals bearing UM-SCC-74A tumors were treated with different doses of BZA. Animals treated with BZA at 8 mg/kg dose showed the maximal tumor growth inhibition (64% at day 27), whereas BZA treatment at 5 mg/kg and 2 mg/kg showed 38% and 13% tumor growth inhibition, respectively, at day 27 (Figure 5A-5B). We next examined the effectiveness of BZA in downregulating pSTAT3 and nanog expression, *in vivo*. BZA treatment at 2 mg/kg did not significantly decrease pSTAT3 (Figure 5C-5D) and nanog expression (Figure 5E-5F). However, BZA treatment at 5 mg/kg and 8 mg/kg doses significantly decreased pSTAT3 and nanog expression in UM-SCC-74A tumors (Figure 5B-5F).

### BZA significantly enhanced the therapeutic efficacy of cisplatin and radiation treatment

**Figure 6:**
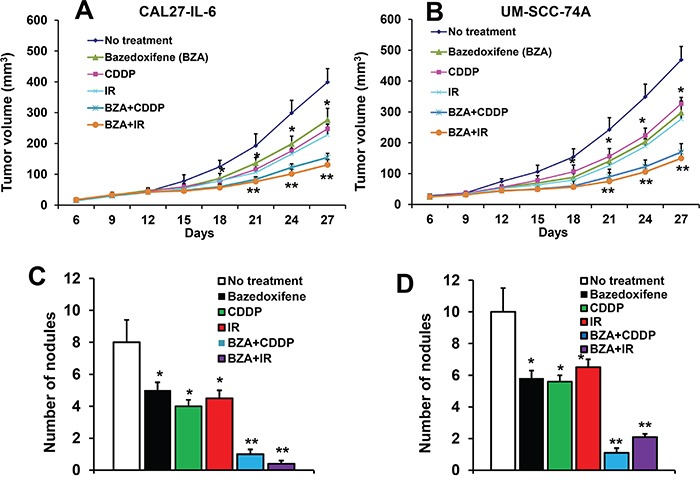
Bazedoxifene significantly decreased tumor growth and tumor metastasis Animals bearing CAL27-IL-6 and UM-SCC-74A tumors were treated with bazedoxifene (BZA, 5 mg/kg/twice a week), cisplatin (CDDP, 5 mg/kg/twice a week) or radiation (IR, 3 Gy/twice a week) either alone or in combination. **A-B**. Tumor growth curves for CAL27-IL-6 (A) and UM-SCC-74 (B). Number of metastatic nodules in animals bearing CAL27-IL-6 **C**. and UM-SCC-74A **D**. *, represents a significant difference (p<0.05) as compared to no treatment group and **, represents a significant difference (p<0.05) as compared to single treatment groups.

Our *in vitro* data suggest that BZA significantly enhanced chemo and radioresistance in head and neck cancer cells. To further validate our *in vitro* results, we performed combination treatment study of BZA with cisplatin (CDDP) and radiation (IR) in a SCID mouse xenograft model. Animals bearing CAL27-IL-6 and treated with BZA (5 mg/kg/twice a week), cisplatin (5 mg/kg/twice a week) and radiation (3 Gy/twice a week) alone showed 34%, 43% and 47% tumor growth reduction, respectively on day 27 (Figure 6A). Combination treatment of BZA with cisplatin and radiation showed 67% and 73% tumor growth reduction, respectively on day 27. We next tested the efficacy of combination treatment in a naturally high IL-6 expressing head and neck cancer cell line (UM-SCC-74A). BZA (5 mg/kg/twice a week), cisplatin (5 mg/kg/twice a week) and radiation (3 Gy/twice a week) alone showed 40%, 33% and 44% tumor growth reduction, respectively on day 27 (Figure 6B). BZA treatment in combination with cisplatin and radiation showed 69% and 72% tumor growth reduction, respectively on day 27.

We next examined the effect of BZA treatment on tumor metastasis. BZA, cisplatin and radiation treatment alone decreased tumor metastasis in CAL27-IL-6 xenograft model by 37%, 50% and 43% and 42%, 44% and 35% in the UM-SCC-74A tumor xenograft groups (Figure 6C-6D). BZA in combination with cisplatin and radiation treatment showed 87% and 92% tumor metastasis reduction in CAL27-IL-6 (Figure 6C) and 89% and 79% tumor metastasis reduction in UM-SCC-74A groups (Figure 6D). In addition, the combination treatment was very well tolerated and it did not cause any animal mortality or induced significant decrease in body weight.

## DISCUSSION

Patients with head and neck cancer encompass a heterogeneous group and even with advancement in treatment options, the overall survival rate for patients with advanced disease has not changed substantially over recent decades [1]. Surgery followed by adjuvant radiotherapy has long been used in the management of patients with HNSCC [55]. More recently, platinum-based regimens are being integrated into the treatment options [56]. However, many of the patients develop resistance to cisplatin, one of the most widely used platinum agents, leading to treatment failure [57, 58]. Therefore, there is an urgent need to develop novel therapeutic strategies that specifically targets pro-survival pathways. Recent studies have shown that IL-6 is one of the main chemokines present in serum samples of cancer patients and elevated IL-6 level is an independent predictor of tumor recurrence, poor survival and tumor metastasis in a number of malignancies including head and neck cancers [6–9, 39, 59]. A number of studies have shown that IL-6 mediates persistent activation of the STAT3 pathway and up regulation of downstream target genes in head and neck cancers leading to increased tumor cell proliferation, migration, survival, invasion, epithelial to mesenchymal transition (EMT), cancer stem cell expansion, and chemoresistance [21, 37, 64]. Therefore, we hypothesized that targeting of IL-6 signaling could reverse the resistant phenotype in tumor cells, thereby enhancing the anti-tumor effects of cisplatin and radiation treatment in HNSCC. To test this hypothesis, we selected bazedoxifene, a newer generation SERM, which has been successfully used in clinics for the treatment of postmenopausal osteoporosis with good safety profile and was recently shown to have a similar structure as Madindoline A (MDL-A) [42–44, 48]. MDL-A is a natural compound that was originally derived from Streptomyces nitrosporeus and has been shown to be a highly selective, non-peptide antagonist of gp130 [60]. MDL-A binds to extracellular domain of gp130 and blocks IL-6-mediated STAT3 activation [61].

In this study, we demonstrate for the first time that bazedoxifene inhibits head and neck cancer growth and metastasis by blocking IL-6 signaling. IL-6 mediates its signaling by binding to its receptor (IL-6R or CD126) and gp130 to form an IL-6/IL-6R/gp130 complex that is clustered into a dimer structure [62]. Our results suggest that bazedoxifene blocks IL-6R/gp130 protein-protein interactions, which leads to the inhibition of downstream signaling mediators including JAK and STAT proteins [63]. In our study, bazedoxifene treatment was very effective in inhibiting tumor cell proliferation, colony formation, tumor cell migration and tumorsphere formation in both the cell lines (CAL27-IL-6 and UM-SCC-74A). UM-SCC-74A contains wild-type p53, whereas CAL27-IL-6 has mutant p53 gene. Interestingly, both these cell lines were equally sensitive to bazedoxifene treatment regardless of p53 mutational status, thereby suggesting that bazedoxifene mediates its anti-tumor effects independent of p53 status. This is important as more that 50% of head and neck tumors have mutant p53 and some of the targeted inhibitors selectively inhibit cell growth in cancer cells with wild-type p53 only [64, 65].

In addition to inhibiting tumor cell proliferation, colony formation and migration, bazedoxifene treatment was also able to reverse chemo and radioresistance in head and neck cancer cells. A number of studies have demonstrated that tumor cells acquire chemo and radio-resistance by increasing the expression of DNA repair proteins and anti-apoptotic proteins (16, 18). Efficient DNA repair in the cancer cells is an important mechanism of therapeutic resistance (39) and inhibition of DNA repair pathway would make tumor cells more sensitive to DNA damaging agents like chemotherapy and radiation treatment [49]. In this study, we have shown for the first time that bazedoxifene can down-regulate the expression of DNA repair proteins XRCC-1 and ERCC-1 in a dose dependent manner. In addition, bazedoxifene treatment also markedly decreased the expression of survivin, a key anti-apoptotic member of IAP family. We have previously shown that survivin levels are markedly upregulated in cisplatin-resistant cells and survivin knockdown with siRNA or treatment with survivin inhibitor YM155 significantly reversed cisplatin resistance in the cisplatin-resistant cells [51]. In addition to reversal of chemo and radioresistance, we have also observed a significant decrease in tumorsphere formation and nanog expression in bazedoxifene treated cells. Nanog is a key stem cell transcription factor that has been shown to be regulated by IL-6 [38, 52]. Nanog is shown to be enriched in cancer stem cell population and has also been shown to mediate cisplatin resistance in cancer cells [66, 67]. In addition, bazedoxifene-mediated downregulation of nanog expression might also be blocking tumor metastasis by decreasing cancer stem cell population [68].

In conclusion, we have shown that bazedoxifene significantly enhances the therapeutic efficacy of chemoradiation treatment by blocking IL-6 signaling. These results from our study provide a scientific rationale to test bazedoxifene in a combination regimen in a clinical trial for the treatment of head and neck cancer patients.

## MATERIALS AND METHODS

### Ethics statement

All animal work was approved by the Ohio State University IACUC Animal ethic committee and conducted according to their guidelines (Animal Welfare Assurance Number A3261-01).

### Cell culture and reagents

The human HNSCC cell line CAL27 was purchased from the ATCC (Manassas, VA, USA). CAL27 is a naturally IL-6 low expressing cell line. IL-6 was overexpressed in CAL27 cells by using a retroviral vector as described previously [18]. UM-SCC-74A, a naturally IL-6 high expressing cell line, was obtained from Dr. Thomas E. Carey (University of Michigan). All tumor cell lines were cultured in DMEM supplemented with 10% fetal bovine serum, non-essential amino acids and penicillin/streptomycin. The identity of all cell lines was confirmed by STR genotyping (Identifier Kit, Applied Biosystems, Carlsband, CA). Bazedoxifene acetate was purchased from (Cayman Chemicals, Ann Arbor, MI) and cisplatin (CDDP) was purchased from Sigma-Aldrich (St. Louis, MO). Antibodies against pSTAT3, pERK1/2, pAkt, Survivin and XRCC-1 were obtained from Cell Signaling Technology (Danvers, MA). Nanog antibody for Western blotting was purchased from Novus Biologicals (Littleton, CO) and for immunohistochemistry was obtained from Abcam (Cambridge, CA). ERCC-1 antibody was purchased from Santa Cruz (Dallas, TX) and pFAK was from Invitrogen (Carlsband, CA). GAPDH antibody was obtained from EMD Millipore (Billerica, MA).

### Cell proliferation assay

The sensitivity of cells to bazedoxifene, cisplatin and radiation was measured using the MTT-based colorimetric cell proliferation kit (Roche Applied Science, Mannheim, Germany) [51]. The percentage cell growth inhibition for each treatment group was calculated by adjusting the untreated control group to 100%.

### Immunoprecipitation

Whole cell lysates were prepared using ice-cold lysis buffer. Cell lysates were precleared by adding protein A/G agarose beads (Pierce Biotechnology, Rockford, IL) and incubating at 4°C for 30 min. gp130 was immunoprecipitated by incubating cell lysates with anti-gp130 antibody (Santa Cruz, Dallas, TX) at 4°C. After overnight incubation with primary antibody, protein A/G agarose slurry was added to cell lysates and further incubated for 4 hours at 4°C with rotary agitation. At the end of incubation, protein A/G beads were washed three times with ice cold lysis buffer and proteins bound to gp130 were collected by boiling the samples in 2x loading buffer.

### Western blot analysis

Whole cell lysates were run on NuPAGE Bis-Tris gels (Invitrogen) under reducing conditions, blotted onto PVDF membranes (GE Healthcare Life Sciences/Amersham, Piscataway, NJ), probed with primary antibodies, then rinsed and incubated with sheep anti-mouse or donkey anti-rabbit conjugated with horseradish peroxidase (GE Healthcare). The membranes were visualized using the ECL western blotting substrate (Pierce, Rockford, IL) according to the manufacturer's instructions.

### Colony formation assay

Cells (2 × 10^3^) were seeded in 3.5 cm dishes and allowed to adhere overnight. Cells were then treated with different concentrations of bazedoxifene and cultured for 10 days. Colonies were fixed with ice-cold methanol and stained with 0.5% crystal violet solution.

### Tumor cell motility assay

Cell motility assay was performed in 6-well plates. A fine scratch in the form of groove was made using a sterile pipette tip in about 90% confluent cells. Cells were then treated different concentrations of bazedoxifene for 48 hours. The migration of cells was monitored microscopically using Nikon Eclipse Ti microscope with DS-Fi1 camera.

### ALDH staining

Tumor cells were cultured in 6 cm dishes and treated with IL-6 and bazedoxifene. At the end of incubation, tumor cells were trypsinized, counted and resuspended in ALDELFLUOR assay buffer (1 × 10^6^/ml, Aldagen Inc, Durham, NC). Each sample was split into two groups; test and control. In the test tube, activated ALDELFLUOR (2.5 μl/0.5ml) was added whereas in the control tubes, 5 μl of DEAB was added first and then activated ALDELFLUOR was added. After incubation at 37°C for 40 min, the cells were washed, resuspended in ALDELFLUOR assay buffer and analyzed by flow cytometry. A minimum of 100,000 events were acquired per sample.

### Tumorsphere forming assays

Tumor cells (300 cells/well) were cultured in 96-well ultralow adhesion culture plates (Corning, PA, USA) containing serum free DMEM/F-12 media supplemented with N2, B-27 (Invitrogen), 10 ng/ml EGF, 10 ng/ml basic-FGF (PeproTech, Rocky Hill, NJ) and antibiotic-antimycotic (Life Technologies, Carlsbad, CA). Cells were treated different concentrations of bazedoxifene and after 10 days, tumorspheres (>50 μm) were counted.

### SCID mouse xenograft model

6-8 week old SCID mice (NCI) were used in all the *in vivo* experiments [50]. Tumor cells (1 × 10^6^) were mixed with 100 μl of Matrigel (BD Biosciences, San Jose, CA) and injected in the flanks of SCID mice. After 8 days, mice were stratified into different groups (n=5), so that the mean tumor volume in each group was comparable. At days 8, 11, 14, 17, 21, 24 animals were treated with bazedoxifene (2, 5 or 8 mg/kg), cisplatin (5 mg/kg) and radiation (3Gy) either alone or in combination. Tumor volume measurements [volume (mm^3^) = L × W^2^/2 (length L, mm; width W, mm)] began on day 6 and continued twice a week until the end of the study. After 27 days, primary tumors and lungs were carefully removed. Primary tumors were analyzed by immunohistochemistry for pSTAT3 and naong expression whereas lungs were examined for metastatic disease.

### Immunohistochemistry

The xenograft tumor tissues and lungs were fixed in 4% paraformaldehyde overnight and paraffin embedded. Tissue sections were deparaffinized and pretreated with antigen retrieval buffer [51]. For nanog staining, endogenous peroxidase and non-specific binding sites were blocked and the sections were incubated with anti-nanog antibody. Primary antibody binding was detected using the reagents from the Vectastain Elite ABC Kit (Vector Laboratories, Burlingame, CA). For pSTAT3 staining, the sections were incubated with primary antibody overnight at 4°C. After washing with PBS, tissue sections were incubated with secondary antibodies (goat anti-rabbit-IgG-Alexa Fluor 594). Tissue sections were then mounted with Prolong gold antifade reagent with DAPI (Invitrogen). The immunohistochemistry images were captured using Nikon Eclipse 80i microscope with DS-Ri1 camera (Nikon, Melville, NY).

### Statistical analysis

Data from all the experiments are expressed as mean ± SEM from a minimum of 3 independent experiments. The data was statistically analyzed by two-way analysis of variance (ANOVA) or Student's t test and a p value of <0.05 was considered significant.
